# Supplementary material for: Obesity, Inflammation, and Mortality in COVID-19: An Observational Study from the Public Health Care System of New York City
Source: J Clin Med. 2022 Jan 26;11(3):622. doi: 10.3390/jcm11030622 (PMC8836690; doi:10.3390/jcm11030622)
Supplement: Supplementary file 1 [file jcm-11-00622-s001.zip › jcm-1534321-supplementary.pdf]

Table S1. Inflammatory markers stratified per BMI group, sex, age group, and survival status

**Marker / Characteristic**	**All Patients**		**BMI group**									
			**BMI < 25**		**25 ≤ BMI < 30**		**30 ≤ BMI < 35**		**35 ≤ BMI < 40**		**BMI ≥ 40**	
	**No. (%)**	**Median (IQR)**	**No. (%)**	**Median (IQR)**	**No. (%)**	**Median (IQR)**	**No. (%)**	**Median (IQR)**	**No. (%)**	**Median (IQR)**	**No. (%)**	**Median (IQR)**
**LDH - U/L**	6476	432.0 (310.0-611.0)	1822 (28.13)	410.5 (289.0-580.0)	2176 (33.6)	434.0 (316.5-612.5)	1313 (20.27)	434.0 (317.0-615.0)	648 (10.01)	465.0 (331.0-666.0)	517 (7.98)	446.0 (334.0-626.0)
**Male**	3997 (61.72)	446 (322-628)	1220 (66.96)	418.0 (291.0-598.5)	1457 (66.96)	444.0 (324.0-624.0)	757 (57.65)	460.0 (339.0-655.0)	318 (49.07)	509.5 (357.0-693.0)	245 (47.39)	466.0 (347.0-672.0)
**Female**	2479 (38.28)	411 (296-580)	602 (33.04)	398.0 (283.0-558.0)	719 (33.04)	419.0 (296.0-580.0)	556 (42.35)	396.0 (296.5-572.5)	330 (50.93)	434.0 (306.0-629.0)	272 (52.61)	434.5 (317.0-605.5)
**Age < 65**	3492 (53.92)	417 (302-595)	736 (40.4)	385.0 (274.5-560.5)	1161 (53.35)	418.0 (300.0-592.0)	807 (61.46)	419.0 (302.0-592.0)	419 (64.66)	456.0 (325.0-629.0)	369 (71.37)	435.0 (330.0-635.0)
**Age ≥ 65**	2984 (46.08)	448 (324-626)	1086 (59.6)	434.5 (296.0-602.0)	1015 (46.65)	457.0 (331.0-631.0)	506 (38.54)	457.0 (347.0-647.0)	229 (35.34)	494.0 (341.0-693.0)	148 (28.63)	463.5 (340.5-615.5)
**Survived**	4615 (71.26)	387 (284-530)	1330 (73)	371.0 (265-509)	1560 (71.69)	393.0 (287.0-532.5)	952 (72.51)	390.0 (295.0-543.0)	442 (68.21)	405.5 (301.0-573.0)	331 (64.02)	391.0 (297.0-543.0)
**Died**	1861 (28.74)	572 (427-794)	492 (27)	553.0 (396-763)	616 (28.31)	582.0 (440.0-792.0)	361 (27.49)	587.0 (426.0-843.0)	206 (31.79)	613.5 (464.0-801.0)	186 (35.98)	551.0 (426.0-788.0)
**Ferritin - ng/mL**	6285	740.0 (337.0-1447.0)	1790 (28.48)	744.8 (337.5-1549.0)	2085 (33.17)	835.2 (395.0-1544.0)	1269 (20.19)	668.0 (308.0-1366.1)	638 (10.15)	659.0 (318.0-1267.6)	503 (8)	574.0 (253.0-1157.0)
**Male**	3891 (61.91)	906 (462-1656)	1193 (66.65)	862.0 (413.1-1688.0)	1403 (67.29)	955.0 (517.7-718.0)	740 (58.31)	911.95 (486.55-1742.35)	313 (49.06)	886.0 (452.5-1514.0)	242 (48.11)	793.5 (383.0-1483.0)
**Female**	2394 (38.09)	492 (219-1013)	597 (33.35)	518.2 (235.0-1141.0)	682 (32.71)	542.5 (236.0-1193.0)	529 (41.69)	463.0 (180.6-847.0)	325 (50.94)	528.1 (263.0-935.1)	261 (51.89)	429.0 (169.0-821.5)
**Age < 65**	3358 (53.43)	746 (307-1424)	723 (40.39)	738.0 (287.0-1553.0)	1100 (52.76)	869.5 (382.25-1575.00)	756 (59.57)	692.0 (278.0-1436.05)	427 (66.93)	674.0 (314.0-1267.6)	352 (69.98)	541.0 (201.5-1051.5)
**Age ≥ 65**	2927 (46.57)	736 (369-1466)	1067 (59.61)	759.5 (361.0-1544.0)	985 (47.24)	796.0 (412.0-1515.0)	513 (40.43)	636.0 (353.0-1268.0)	211 (33.07)	650.3 (321.0-1269.0)	151 (30.02)	612.8 (321.0-1245.0)
**Survived**	4508 (71.73)	625 (282-1241)	1318 (73.63)	647.0 (291.0-1287.0)	1500 (71.94)	746.95 (336.5-1349.55)	914 (72.03)	569.0 (249.0-1169.0)	447 (70.06)	572.0 (272.0-1106.0)	329 (65.41)	431.0 (179.3-896.0)
**Died**	1777 (28.27)	1043 (558-1952)	472 (26.37)	1063.5 (531.6-2000.0)	585 (28.06)	1112.2 (654.0-2000.0)	355 (27.97)	1090.8 (542.0-1951.0)	191 (29.94)	875.9 (515.0-1532.0)	174 (34.59)	833.5 (479.0-1527.0)
**CRP - mg/dL**	3114	24.22 (8.40-92.80)	897 (28.81)	19.57 (6.56-86.10)	997 (32.02)	26.82 (10.09-111.81)	614 (19.72)	23.01 (8.61-97.15)	327 (10.5)	28.44 (8.50-95.10)	279 (8.96)	22.99 (9.51-69.10)
**Male**	1940 (62.3)	26.85 (9.76-105.84)	613 (68.34)	21.44 (7.42-91.96)	667 (66.9)	28.73 (11.79-118.60)	361 (58.79)	27.09 (9.81-19.30)	173 (52.91)	31.18 (11.15-93.60)	126 (45.16)	26.07 (9.90-84.02)
**Female**	1174 (37.7)	19.52 (6.97-76.60)	284 (31.66)	15.13 (4.95-61.70)	330 (33.1)	20.17 (6.97-90.10)	253 (41.21)	18.67 (6.97-73.60)	154 (47.09)	25.44 (7.96-95.60)	153 (54.84)	22.05 (8.98-61.00)
**Age < 65**	1757 (56.42)	25.30 (8.01-93.40)	407 (45.37)	21.00 (5.64-90.80)	553 (55.47)	27.62 (9.40-106.90)	389 (63.36)	24.97 (8.06-111.64)	216 (66.06)	28.70 (3.14-92.55)	192 (68.82)	22.10 (8.54-63.20)
**Age ≥ 65**	1357 (43.58)	22.64 (8.98-91.70)	490 (54.63)	19.13 (6.90-84.40)	444 (44.53)	25.70 (10.72-118.75)	225 (36.64)	20.83 (9.71-81.00)	111 (33.94)	25.64 (8.88-96.30)	87 (31.18)	25.15 (11.87-80.90)
**Survived**	2294 (73.67)	20.81 (6.92-83.60)	679 (75.7)	15.80 (5.28-69.59)	748 (75.03)	24.14 (8.06-98.69)	451 (73.45)	19.51 (7.19-82.33)	229 (70.03)	27.02 (6.91-101.26)	187 (67.03)	21.56 (8.34-64.90)
**Died**	820 (26.33)	29.70 (14.20-141.20)	218 (24.3)	30.01 (11.97-148.00)	249 (24.97)	31.80 (18.29-151.70)	163 (26.55)	28.88 (15.17-156.90)	98 (29.97)	29.48 (14.28-62.80)	92 (32.97)	25.42 (11.24-88.11)

**IL-6 - pg/mL**	2237	82.10 (35.50-174.30)	596 (26.64)	77.45 (33.55-175.15)	720 (32.19)	91.25 (39.60-191.25)	472 (21.1)	76.50 (34.55-156.85)	248 (11.09)	80.20 (36.30-188.15)	201 (8.99)	85.10 (32.30-164.00)
**Male**	1420 (63.48)	90.4 (39.9-193.7)	408 (68.46)	80.1 (36.1-191.1)	497 (69.03)	101.1 (44.6-207.1)	291 (61.65)	80.6 (39.8-177.6)	120 (48.39)	99.0 (38.0-215.4)	104 (51.74)	87.0 (35.2-171.6)
**Female**	817 (36.52)	71.0 (29.0-155.3)	188 (31.54)	72.1 (26.5-150.2)	223 (30.97)	69.5 (31.6-162.0)	181 (38.35)	72.4 (26.5-127.2)	128 (51.61)	64.0 (31.7-170.4)	97 (48.26)	81.1 (29.0-158.6)
**Age < 65**	1212 (54.18)	77.2 (32.2-168.0)	235 (39.43)	68.5 (30.4-173.7)	386 (53.61)	86.2 (36.9-175.2)	280 (59.32)	68.3 (30.6-148.5)	163 (65.73)	80.4 (34.9-187.4)	148 (73.63)	66.3 (27.5-148.3)
**Age ≥ 65**	1025 (45.82)	91.0 (39.4-189.4)	361 (60.57)	84.0 (36.0-177.6)	334 (46.39)	96.3 (44.7-208.8)	192 (40.68)	83.8 (39.1-161.0)	85 (34.27)	80.0 (43.3-191.6)	53 (26.37)	137.2 (65.9-16.3)
**Survived**	1391 (62.18)	59.0 (24.0-115.9)	398 (66.78)	57.3 (23.0-116.5)	451 (62.64)	63.3 (24.0-127.7)	294 (62.29)	57.0 (24.5-101.1)	142 (57.26)	59.3 (29.5-103.7)	106 (52.74)	51.4 (18.2-96.6)
**Died**	846 (37.82)	141.3 (75.4-289.4)	198 (33.22)	136.2 (64.7-296.0)	269 (37.36)	151.6 (85.6-341.0)	178 (37.71)	135.9 (76.0-243.2)	106 (42.74)	148.4 (66.0-291.5)	95 (47.26)	136.7 (65.8-245.2)
**D-dimer - mg/mL**	6471	598.0 (304.0-1715.0)	1844 (28.5)	766.0 (365.5-2371.5)	2197 (33.95)	565.0 (299.0-1357.0)	1286 (19.87)	528.0 (279.0-1333.0)	624 (9.64)	523.5 (287.0-1105.5)	520 (8.04)	484.0 (261.0-1079.0)
**Male**	3993 (61.71)	569 (290-1796)	1231 (66.76)	730 (347-2511)	1467 (66.77)	546 (286-1411)	744 (57.85)	488 (258-1303)	310 (49.68)	444 (243-977)	241 (46.35)	488 (250-1336)
**Female**	2478 (38.29)	647 (329-1592)	613 (33.24)	792 (405-2182)	730 (33.23)	610 (326-1289)	542 (42.15)	626 (320-1468)	314 (50.32)	623 (320-1252)	279 (53.65)	481 (272-1038)
**Age < 65**	3441 (53.18)	462 (352-1071)	739 (40.08)	568 (280-1591)	1154 (52.53)	446 (253-1038)	780 (60.65)	426 (244-1052)	398 (63.78)	441 (250-902)	370 (71.15)	426 (239-935)
**Age ≥ 65**	3030 (46.82)	770 (406-2378)	1105 (59.92)	883 (448-2846)	1043 (47.47)	727 (394-2092)	506 (39.35)	694 (379-2222)	226 (36.22)	710 (364-2233)	150 (28.85)	647 (371-1702)
**Survived**	4621 (71.41)	507 (271-1109)	1351 (73.26)	643 (319-1850)	1580 (71.92)	497 (265-1018)	926 (72.01)	442 (250-977)	427 (68.43)	453 (252-951)	337 (64.81)	427 (237-872)
**Died**	1850 (28.59)	912 (449-3395)	493 (26.74)	1204 (589-3698)	617 (28.08)	911 (433-3680)	360 (27.99)	913 (450-3146)	197 (31.57)	636 (397-2616)	183 (35.19)	678 (341-2194)
**N/L**	8795	5.74 (3.40-9.93)	2574 (29.27)	6.37 (3.53-11.33)	2963 (33.69)	5.98 (3.53-9.97)	1745 (19.84)	5.35 (3.30-8.99)	832 (9.46)	5.26 (3.21-8.86)	681 (7.74)	4.77 (3.01-7.81)
**Male**	5218 (59.33)	6.40 (3.77-10.95)	1689 (65.62)	7.11 (3.84-12.27)	1910 (64.46)	6.46 (3.83-10.79)	943 (54.04)	6.08 (3.64-10.29)	384 (46.15)	5.82 (3.48-10.19)	292 (42.88)	5.45 (3.64-8.57)
**Female**	3577 (40.67)	4.94 (3.02-8.39)	885 (34.38)	5.43 (3.16-9.68)	1053 (35.54)	5.09 (3.09-8.53)	802 (45.96)	4.61 (2.91-7.34)	448 (53.85)	4.89 (3.11-7.71)	389 (57.12)	4.29 (2.83-7.38)
**Age < 65**	4839 (55.02)	5.21 (3.12-14.61)	1088 (42.27)	5.58 (3.06-10.07)	1630 (55.01)	5.62 (3.37-9.62)	1089 (62.41)	5.05 (3.11-8.40)	551 (66.23)	4.80 (2.99-7.86)	481 (70.63)	4.45 (2.90-7.24)
**Age ≥ 65**	3956 (44.98)	6.43 (3.79-11.05)	1486 (57.73)	7.04 (3.95-12.09)	1333 (44.99)	6.33 (3.78-10.70)	656 (37.59)	5.91 (3.52-9.96)	281 (33.77)	6.26 (4.04-10.73)	200 (29.37)	5.63 (3.59-9.44)
**Survived**	6537 (74.33)	5.14 (3.10-8.75)	1933 (75.1)	5.49 (3.20-9.93)	2221 (74.96)	5.41 (3.25-9.04)	1317 (75.47)	4.86 (3.02-7.90)	597 (71.75)	4.75 (2.96-7.60)	469 (68.87)	4.30 (2.74-6.57)
**Died**	2258 (25.67)	8.02 (4.83-12.88)	641 (24.9)	9.55 (5.80-16.00)	742 (25.04)	7.77 (4.95-12.23)	428 (24.53)	7.77 (4.65-11.80)	235 (28.25)	7.38 (4.11-12.19)	212 (31.13)	6.23 (4.06-10.12)
**L/CRP**	3109	3.98 (1.07-12.32)	896 (28.82)	4.64 (1.05-16.17)	995 (32)	3.46 (0.91-10.16)	613 (19.72)	4.11 (1.10-12.81)	327 (10.52)	3.14 (1.22-13.75)	278 (8.94)	4.61 (1.69-11.61)
**Male**	1936 (62.27)	3.27 (0.91-10.09)	612 (68.3)	3.87 (0.88-12.37)	666 (66.93)	2.89 (0.82-8.01)	360 (58.73)	3.33 (0.89-11.34)	173 (52.91)	2.48 (1.02-10.39)	125 (44.96)	4.26 (1.43-11.63)
**Female**	1173 (37.73)	5.40 (1.57-17.03)	284 (31.7)	6.53 (1.89-25.90)	329 (33.07)	4.99 (1.26-16.73)	253 (41.27)	5.45 (1.76-16.55)	154 (47.09)	4.63 (1.39-17.54)	153 (55.04)	4.83 (2.09-11.43)
**Age < 65**	1753 (56.38)	3.98 (1.05-13.01)	406 (45.31)	4.66 (0.95-18.37)	552 (55.48)	3.45 (0.92-10.82)	388 (63.3)	4.03 (0.98-14.08)	216 (66.06)	3.13 (1.22-15.47)	191 (68.71)	5.19 (1.77-12.43)
**Age ≥ 65**	1356 (43.62)	3.98 (1.10-11.38)	490 (54.69)	4.56 (1.19-15.12)	443 (44.52)	3.46 (0.87-9.70)	225 (36.7)	4.23 (1.28-11.75)	111 (33.94)	3.23 (1.21-10.65)	87 (31.29)	3.98 (1.35-8.80)
**Survived**	2290 (73.66)	4.77 (1.26-16.17)	678 (75.67)	5.71 (1.36-22.61)	747 (75.08)	4.19 (1.06-13.20)	450 (73.41)	5.26 (1.38-16.10)	229 (70.03)	3.37 (1.17-16.75)	186 (66.91)	5.24 (1.84-13.10)
**Died**	819 (26.34)	2.73 (0.68-6.50)	218 (24.33)	3.03 (0.55-7.01)	248 (24.92)	2.50 (0.62-5.21)	163 (26.59)	2.73 (0.67-5.80)	98 (29.97)	2.44 (1.44-6.50)	92 (33.09)	3.15 (0.87-8.16)

Notes: (1) The first available laboratory values after presentation were taken into account in this analysis if within 24 hours from presentation, (2) BMI in kg/m^2; **Abbreviations**: BMI = body mass index, IQR = interquartile range, no. = number, ng = nanogram, mg = milligram, L = liter, dL = deciliter, mL = milliliter, U = unit.

Supplementary Table S2. Baseline patient characteristics: Univariate and multivariate logistic regression analysis for the outcome of invasive mechanical ventilation after excluding patients that met the outcome of mortality

Variable	Univariate analysis	Multivariate Analysis			
		**Model A N = 6567**	**Model B N = 6567**	**Model C N = 6567**	**Model D N = 6567**
	**OR, 95% CI, *p*-value**	**OR, 95% CI, *p*-value**	**OR, 95% CI, *p*-value**	**OR, 95% CI, *p*-value**	**OR, 95% CI, *p*-value**
**Male sex**	1.512 (1.303-1.756) *p* <0.001	1.407 (1.082-1.829) *p* = 0.011	1.392 (1.072-1.808) *p* = 0.013	1.382 (1.065-1.793) *p* = 0.015	1.395 (1.076-1.809) *p* = 0.012
**Age (All)**	1.005 (1.001-1.009) *p* = 0.009				
**<50**	1.033 (1.012-1.055) *p* = 0.002	*	*	*	*
**50-64**	1.001 (0.973-1.031) *p* = 0.934	1.345 (0.982-1.840) *p* = 0.065	1.322 (0.966-1.809) *p* = 0.081	1.320 (0.965-1.806) *p* = 0.082	1.340 (0.979-1.834) *p* = 0.067
**65-74**	1.047 (0.996-1.101) *p* = 0.069)	1.645 (1.144-2.366) *p* = 0.007	1.597 (1.111-2.294) *p* = 0.011	1.588 (1.106-2.280) *p* = 0.012	1.622 (1.130-2.330) *p* = 0.009
**≥75**	0.959 (0.932-0.986) *p* = 0.003	0.773 (0.481-1.241) *p* = 0.286	0.738 (0.461-1.182) *p* = 0.207	0.728 (0.456-1.164) *p* = 0.185	0.751 (0.470-1.198) *p* = 0.229
**Ethnicity/Race**					
**Hispanic/Latino**	1.329 (1.017-1.736) *p* = 0.037	1.216 0.759 1.947) *p* = 0.417	1.208 (0.755-1.934) *p* = 0.431	1.209 (0.755-1.935) *p* = 0.429	1.226 (0.766-1.963) *p* = 0.396
**Non-Hispanic Black**	0.842 (0.633-1.120) *p* = 0.237	0.713 (0.432-1.175) *p* = 0.184	0.718 (0.436-1.183) *p* = 0.193	0.716 (0.435-1.180) *p* = 0.190	0.714 (0.433-1.176) *p* = 0.186
**Non-Hispanic White**	*	*	*	*	*
**Asian**	1.829 (1.275-2.622) *p* = 0.001	0.839 (0.397-1.775) *p* = 0.647	0.813 (0.385-1.716) *p* = 0.587	0.799 (0.379-1.686) *p* = 0.557	0.820 (0.389-1.730) *p* = 0.602
**Other/Unknown**	1.474 (1.100-1.976) *p* = 0.009	1.044 (0.617-1.767) *p* = 0.872	1.042 (0.616-1.763) *p* = 0.877	1.040 (0.615-1.759) *p* = 0.882	1.048 (0.620-1.772) *p* = 0.861
**BMI < 25 (reference)**	0.703 (0.596-0.828) *p* <0.001	*			
**25 ≤ BMI < 30**	1.036 (0.894-1.201) *p* = 0.636	1.174 (0.857-1.608) *p* = 0.318			
**30 ≤ BMI < 35**	1.008 (0.846-1.201) *p* = 0.926	1.236 (0.863-1.769) *p* = 0.247			
**35 ≤ BMI < 40**	1.167 (0.929-1.465) *p* = 0.185	0.996 (0.605-1.640) *p* = 0.987			
**BMI ≥ 30**	1.289 (1.119-1.486) *p* <0.001		1.161 (0.899-1.498) *p* = 0.252		
**BMI ≥ 35**	1.461 (1.233-1.731) *p* <0.001			1.173 (0.849-1.621) *p* = 0.333	
**BMI ≥ 40**	1.707 (1.367-2.132) *p* <0.001	1.824 (1.143-2.911) *p* = 0.012			1.629 (1.078-2.463) *p* = 0.021
**Hypertension**	0.971 (0.844-1.117) *p* = 0.681	1.373 (0.987-1.910) *p* = 0.060	1.403 (1.010-1.950) *p* = 0.044	1.405 (1.011-1.952) *p* = 0.043	1.385 (0.997-1.924) *p* = 0.052
**Hyperlipidemia**	0.884 (0.747-1.047) *p* = 0.154	0.754 (0.544-1.045) *p* = 0.090	0.746 (0.539-1.033) *p* = 0.078	0.748 (0.540-1.036) *p* = 0.081	0.756 (0.546-1.047) *p* = 0.092
**Diabetes**	0.851 (0.727-0.997) *p* = 0.046	0.941 (0.663-1.336) *p* = 0.734	0.936 (0.660-1.327) *p* = 0.710	0.935 (0.659-1.325) *p* = 0.705	0.930 (0.656-1.319) *p* = 0.684
**Heart failure**	0.760 (0.577-1.001) *p* = 0.051	0.900 (0.529-1.531) *p* = 0.697	0.909 (0.535-1.546) *p* = 0.725	0.906 (0.533-1.541) *p* = 0.717	0.893 (0.525-1.519) *p* = 0.676
**CAD**	0.707 (0.536-0.933) *p* = 0.014	0.463 (0.244-0.878) *p* = 0.018	0.464 (0.244-0.880) *p* = 0.019	0.464 (0.245-0.881) *p* = 0.019	0.466 (0.246-0.884) *p* = 0.020
**PAD**	0.681 (0.401-1.156) *p* = 0.155	1.215 (0.514-2.870) *p* = 0.658	1.207 (0.511-2.849) *p* = 0.668	1.202 (0.509-2.837) *p* = 0.675	1.207 (0.511-2.851) *p* = 0.669
**Afib**	0.764 (0.553-1.056) *p* = 0.103	0.894 (0.469-1.704) *p* = 0.733	0.896 (0.470-1.706) *p* = 0.738	0.898 (0.472-1.711) *p* = 0.744	0.897 (0.471-1.709) *p* = 0.742
**Stroke**	1.111 (0.962-1.282) *p* = 0.153	0.577 (0.430-0.776) *p* <0.001	0.582 (0.433-0.781) *p* = <0.001	0.582 (0.433-0.782) *p* <0.001	0.581 (0.432-0.781) *p* <0.001

**CKD**	0.943 (0.757-1.173) *p* = 0.597	1.069 (0.709-1.612) *p* = 0.750	1.054 (0.699-1.589) *p* = 0.801	1.046 (0.694-1.576) *p* = 0.830	1.056 (0.701-1.592) *p* = 0.794
**COPD**	0.853 (0.444-1.636) *p* = 0.632	1.045 (0.369-2.961) *p* = 0.935	1.010 (0.357-2.861) *p* = 0.985	1.007 (0.355-2.861) *p* = 0.990	1.021 (0.361-2.892) *p* = 0.968
**Asthma**	0.934 (0.739-1.182) *p* = 0.571	0.884 (0.580-1.346) *p* = 0.565	0.916 (0.604-1.390) *p* = 0.681	0.914 (0.602-1.389) *p* = 0.674	0.882 (0.580-1.342) *p* = 0.558
**HIV/AIDS**	1.172 (0.762-1.803) *p* = 0.471	1.443 (0.774-2.798) *p* = 0.278	1.414 (0.730-2.739) *p* = 0.305	1.414 (0.730-2.739) *p* = 0.305	1.418 (0.732-2.746) *p* = 0.301
**Liver cirrhosis**	0.789 (0.425-1.467) p = 0.454	0.322 (0.078-1.322) *p* = 0.116	0.318 (0.077-1.306) *p* = 0.112	0.318 (0.077-1.306) *p* = 0.112	0.322 (0.078-1.324) *p* = 0.116

Notes: (1) BMI in kg/m2, (2) age in years, (3) Model A includes the variables that were found to have significant univariate association and BMI as ordinal variable: BMI < 25, 25 ≤ BMI < 30, 30 ≤ BMI < 35, 35 ≤ BMI < 40, and BMI ≥ 40, (4) Model B includes the variables that were found to have significant univariate association and BMI as dichotomous variable: BMI < 30 and BMI ≥ 30, (5) Model C includes the variables that were found to have significant univariate association and BMI as dichotomous variable: BMI < 35 and BMI ≥ 35, (6) Model D includes the variables that were found to have significant univariate association and BMI as dichotomous variable: BMI < 40 and BMI ≥ 40.

Abbreviations: BMI = body mass index, OR = odds ratio, CI = confidence interval, kg = kilogram, m = meter, CAD = coronary artery disease, PAD = peripheral artery disease, Afib = atrial fibrillation, CKD = chronic kidney disease, COPD = chronic obstructive pulmonary disease, HIV = human immunodeficiency, AIDS = acquired immunodeficiency syndrome.

Supplementary Table S3. Baseline patient characteristics: Univariate and multivariate logistic regression analysis for the outcome of admission to the intensive care unit after excluding patients that met the outcome of mortality

Variable	Univariate analysis	Multivariate Analysis			
		**Model A N = 6567**	**Model B N = 6567**	**Model C N = 6567**	**Model D N = 6567**
	**OR, 95% CI, *p*-value**	**OR, 95% CI, *p*-value**	**OR, 95% CI, *p*-value**	**OR, 95% CI, *p*-value**	**OR, 95% CI, *p*-value**
**Male sex**	1.672 (1.497-1.867) *p* <0.001	1.461 (1.247-1.713) *p* <0.001	1.456 (1.243-1.706) *p* <0.001	1.464 (1.250-1.714) *p* <0.001	1.451 (1.240-1.698) *p* <0.001
**Age (All)**	0.998 (0.995-1.001) *p* = 0.248				
**<50**	1.037 (1.023-1.052) *p* <0.001	*	*	*	*
**50-64**	1.007 (0.986-1.029) *p* = 0.503	1.175 (0.973-1.419) *p* = 0.095	1.169 (0.968-1.411) *p* = 0.105	1.173 (0.971-1.416) *p* = 0.098	1.175 (0.973-1.419) *p* = 0.094
**65-74**	1.001 (0.964-1.040) *p* = 0.950	0.972 (0.771-1.225) *p* = 0.808	0.968 (0.769-1.219) *p* = 0.785	0.975 (0.775-1.227) *p* = 0.830	0.973 (0.773-1.225) *p* = 0.818
**≥75**	0.941 (0.920-0.963) *p* <0.001	0.616 (0.469-0.808) *p* <0.001	0.614 (0.469-0.804) *p* <0.001	0.622 (0.475-0.814) *p* <0.001	0.616 (0.471-0.805) *p* <0.001
**Ethnicity/Race**					
**Hispanic/Latino**	1.127 (0.929-1.367) *p* = 0.225	0.095 (0.684 1.197) *p* = 0.484	0.893 (0.676-1.181) *p* = 0.429	0.896 (0.678-1.185) *p* = 0.441	0.901 (0.681-1.191) *p* = 0.462
**Non-Hispanic Black**	0.896 (0.733-1.096) *p* = 0.286	0.712 (0.532-0.953) *p* = 0.022	0.714 (0.534-0.956) *p* = 0.024	0.710 (0.531-0.950) *p* = 0.021	0.714 (0.533-0.955) *p* = 0.023
**Non-Hispanic White**	*	*	*	*	*
**Asian**	1.902 (1.456-2.484) *p* <0.001	1.698 (1.164-2.475) *p* = 0.006	1.691 (1.161-2.464) *p* = 0.006	1.696 (1.165-2.470) *p* = 0.006	1.690 (1.161-2.460) *p* = 0.006
**Other/Unknown**	1.384 (1.119-1.712) *p* = 0.003	1.025 (0.752-1.396) *p* = 0.877	1.019 (0.748-1.389) *p* = 0.903	1.020 (0.749-1.389) *p* = 0.902	1.021 (0.750-1.391) *p* = 0.894
**BMI < 25 (reference)**	0.746 (0.662-0.840) *p* <0.001	*			
**25 ≤ BMI < 30**	0.922 (0.826-1.030) *p* = 0.151	0.903 (0.749-1.088) *p* = 0.281			
**30 ≤ BMI < 35**	1.139 (1.003-1.294) *p* = 0.044	0.949 (0.764-1.179) *p* = 0.637			

**35 ≤ BMI < 40**	1.296 (1.096-1.532) *p* = 0.002	1.100 (0.834-1.451) *p* = 0.501			
**BMI ≥ 30**	1.378 (1.240-1.531) *p* <0.001		1.093 (0.934-1.279) *p* = 0.269		
**BMI ≥ 35**	1.438 (1.263-1.636) *p* <0.001			1.219 (0.999-1.487) *p* = 0.051	
**BMI ≥ 40**	1.496 (1.253-1.787) *p* <0.001	1.226 (0.905-1.662) *p* = 0.189			1.270 (0.971-1.678) *p* = 0.092
**Hypertension**	0.967 (0.872-1.073) *p* = 0.531	1.143 (0.939-1.390) *p* = 0.182	1.146 (0.943-1.394) *p* = 0.172	1.138 (0.936-1.384) *p* = 0.195	1.142 (0.939-1.389) *p* = 0.182
**Hyperlipidemia**	0.926 (0.818-1.047) *p* = 0.219	0.894 (0.738-1.083) *p* = 0.251	0.890 (0.735-1.078) *p* = 0.234	0.890 (0.735-1.077) *p* = 0.231	0.896 (0.739-1.084) *p* = 0.259
**Diabetes**	0.931 (0.827-1.049) *p* = 0.242	1.255 (1.014-1.554) *p* = 0.036	1.267 (1.024-1.567) *p* = 0.029	1.266 (1.024-1.566) *p* = 0.030	1.263 (1.021-1.562) *p* = 0.032
**Heart failure**	0.802 (0.659-0.976) *p* = 0.028	0.877 (0.650-1.185) *p* = 0.394	0.889 (0.658-1.199) *p* = 0.440	0.885 (0.655-1.194) *p* = 0.423	0.881 (0.653-1.190) *p* = 0.410
**CAD**	0.939 (0.781-1.129) *p* = 0.503	1.041 (0.779-1.391) *p* = 0.786	1.029 (0.770-1.375) *p* = 0.846	1.036 (0.775-1.384) *p* = 0.812	1.030 (0.771-1.377) *p* = 0.351
**PAD**	0.757 (0.525-1.092) *p* = 0.136	0.934 (0.560-1.558) *p* = 0.795	0.937 (0.562-1.561) *p* = 0.803	0.934 (0.560-1.557) *p* = 0.794	0.939 (0.564-1.566) *p* = 0.810
**Afib**	1.271 (1.036-1.560) *p* = 0.022	1.710 (1.248-2.343) *p* <0.001	1.712 (1.250-2.345) *p* <0.001	1.704 (1.244-2.335) *p* <0.001	1.714 (1.251-2.347) *p* <0.001
**Stroke**	0.804 (0.720-0.897) *p* <0.001	0.529 (0.442-0.633) *p* <0.001	0.527 (0.441-0.631) *p* <0.001	0.527 (0.441-0.631) *p* <0.001	0.527 (0.441-0.631) *p* <0.001
**CKD**	0.993 (0.846-1.164) *p* = 0.926	1.120 (0.880-1.424) *p* = 0.358	1.123 (0.883-1.428) *p* = 0.346	1.122 (0.882-1.427) *p* = 0.347	1.121 (0.882-1.426) *p* = 0.351
**COPD**	0.710 (0.428-1.176) *p* = 0.183	0.798 (0.404-1.576) *p* = 0.516	0.804 (0.407-1.588) *p* = 0.530	0.807 (0.408-1.593) *p* = 0.536	0.807 (0.409-1.594) *p* = 0.538
**Asthma**	0.792 (0.661-0.948) *p* = 0.011	0.784 (0.604-1.018) *p* = 0.068	0.801 (0.618-1.038) *p* = 0.093	0.788 (0.608-1.022) *p* = 0.073	0.790 (0.609-1.025) *p* = 0.076
**HIV/AIDS**	1.027 (0.734-1.437) *p* = 0.876	1.179 (0.759-1.831) *p* = 0.463	1.192 (0.768-1.850) *p* = 0.434	1.196 (0.771-1.857) *p* = 0.424	1.188 (0.766-1.843) *p* = 0.442
**Liver cirrhosis**	0.661 (0.410-1.064) p = 0.089	0.607 (0.321-1.149) *p* = 0.126	0.603 (0.319 1.141) *p* = 0.120	0.606 (0.320-1.145) *p* = 0.123	0.605 (0.320-1.145) *p* = 0.123

Notes: (1) BMI in kg/m2, (2) age in years, (3) Model A includes the variables that were found to have significant univariate association and BMI as ordinal variable: BMI < 25, 25 ≤ BMI < 30, 30 ≤ BMI < 35, 35 ≤ BMI < 40, and BMI ≥ 40, (4) Model B includes the variables that were found to have significant univariate association and BMI as dichotomous variable: BMI < 30 and BMI ≥ 30, (5) Model C includes the variables that were found to have significant univariate association and BMI as dichotomous variable: BMI < 35 and BMI ≥ 35, (6) Model D includes the variables that were found to have significant univariate association and BMI as dichotomous variable: BMI < 40 and BMI ≥ 40.

Abbreviations: BMI = body mass index, OR = odds ratio, CI = confidence interval, kg = kilogram, m = meter, CAD = coronary artery disease, PAD = peripheral artery disease, Afib = atrial fibrillation, CKD = chronic kidney disease, COPD = chronic obstructive pulmonary disease, HIV = human immunodeficiency, AIDS = acquired immunodeficiency syndrome.

Supplementary Table S4. Univariate association of timing of admission with mortality

**Quantile**	**All patients (N = 8833)**		
	**No.**	**OR, 95% CI**	**p-Value**
**1st quantile (3/3 - 3/23/2020)**	883	*	
**2nd quantile (3/23 - 3/27/2020)**	883	1.163 (0.945-1.431)	0.154
**3rd quantile (3/27 - 3/31/2020)**	883	1.355 (0.104-1.662)	0.004
**4th quantile (3/31 - 4/3/2020)**	883	1.418 (1.156-1.739)	<0.001
**5th quantile (4/3 - 4/8/2020)**	883	1.668 (1.363-2.041)	<0.001
**6th quantile (4/8 - 4/12/2020)**	883	1.307 (1.064-1.604)	0.011
**7th quantile (4/12 - 4/18/2020)**	883	1.071 (0.869-1.320)	0.522
**8th quantile (4/18 - 4/30/2020)**	884	0.731 (0.586-0.910)	0.005
**9th quantile (4/30 - 6/2/2020)**	884	0.254 (0.192-0.336)	<0.001
**10th quantile (6/3 - 10/31/2020)**	884	0.151 (0.108-0.210)	<0.001
**1st quartile (3/3 - 3/29/2020)**	2209	*	

**2nd quartile (3/29 - 4/8/2020)**	2208	1.335 (1.177-1.515)	<0.001
**3rd quartile (4/8 - 4/23/2020)**	2208	0.992 (0.871-1.129)	0.902
**4th quartile (4/23 - 10/31/2020)**	2208	0.241 (0.203-0.286)	<0.001

Notes: (1) N = 8833, (2) BMI in kg/m^2; **Abbreviations:** BMI = body mass index, OR = odds ratio, CI = confidence interval, kg = kilogram, m = meter.

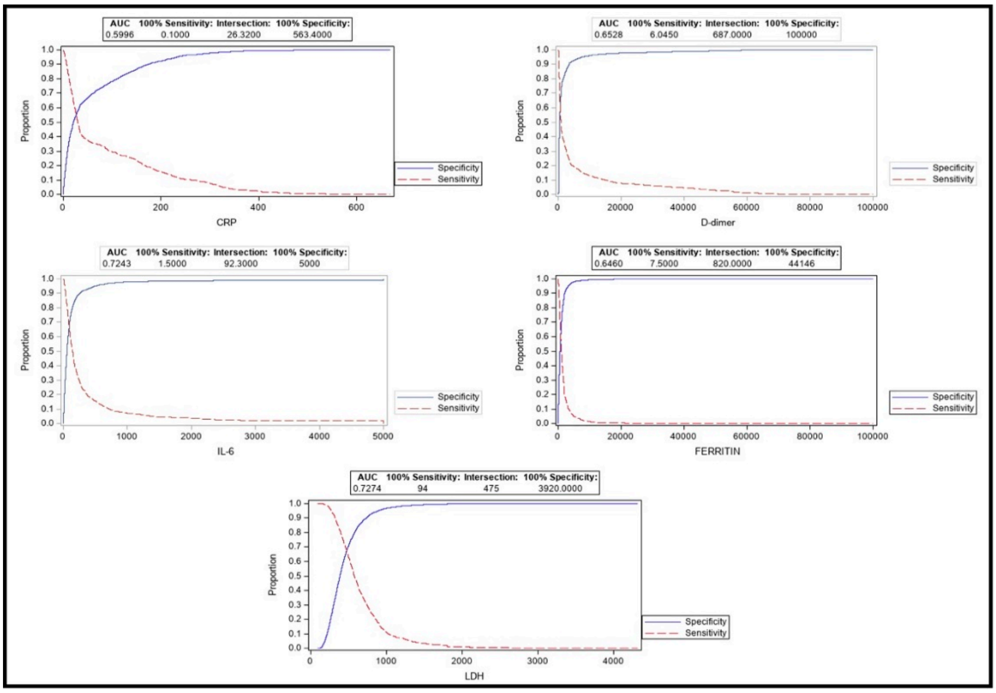

Figure S1. Inflammatory markers: Operating characteristics for mortality; **Notes:** LDH in U/L, ferritin in ng/mL, CRP in mg/dL, d-dimer in mg/mL, IL-6 in pg/mL.

Abbreviations: AUC = area under the curve, CRP = C-reactive protein, IL-6 = interleukin-6, LDH = lactic dehydrogenase

Supplementary Table S5. Inflammatory markers: Subgroup analysis based on BMI of the multivariate logistic regression analysis results for the outcome of mortality

Panel A (N = 5068)					
	**Multivariate analysis**				
**Variable**	**BMI < 25 N = 1428 (28.18%) OR, 95% CI, *p*-value**	**25 ≤ BMI < 30 N = 1703 (33.60%) OR, 95% CI, *p*-value**	**30 ≤ BMI < 35 N = 1011 (19.95%) OR, 95% CI, *p*-value**	**35 ≤ BMI < 40 N = 516 (10.18%) OR, 95% CI, *p*-value**	**BMI ≥ 40 N = 410 (8.09%) OR, 95% CI, *p*-value**
Age < 65	0.322 (0.240-0.433) *p* <0.001	0.384 (0.302-0.489) *p* <0.001	0.409 (0.299-0.558) *p* <0.001	0.630 (0.414-0.960) *p* = 0.031	0.443 (0.270-0.726) *p* = 0.001
Age ≥ 65	3.102 (2.310-4.166) *p* <0.001	2.602 (2.047-3.307) *p* <0.001	2.446 (1.791-3.340) *p* <0.001	1.587 (1.042-2.417) *p* = 0.031	2.257 (1.377-3.698) *p* = 0.001
Male sex	1.008 (0.762-1.333) *p* = 0.955	1.292 (0.999-1.672) *p* = 0.051	1.534 (1.112-2.116) *p* = 0.009	1.211 (0.809-1.812) *p* = 0.353	1.087 (0.678-1.742) *p* = 0.728
WBC -10^3/μL	1.782 (0.554-1.102) *p* = 0.160	0.856 (0.594-1.235) *p* = 0.407	0.780 (0.418-1.457) *p* = 0.436	0.688 (0.245-1.928) *p* = 0.477	1.501 (0.673-3.347) *p* = 0.321
Neutrophils - 10^3/μL	1.340 (0.934-1.922) *p* = 0.112	1.231 (0.844-1.795) *p* = 0.280	1.366 (0.714-2.615) *p* = 0.346	1.457 (0.507-4.185) *p* = 0.484	0.650 (0.284-1.488) *p* = 0.308
Monocytes - 10^3/μL	0.999 (0.609-1.641) *p* = 0.998	0.523 (0.288-0.949) *p* = 0.033	0.795 (0.337-1.877) *p* = 0.600	1.124 (0.288-4.389) *p* = 0.866	0.492 (0.153-1.577) *p* = 0.233
Lymphocytes - 10^3/μL	1.255 (0.875-1.801) *p* = 0.217	1.203 (0.828-1.748) *p* = 0.332	1.331 (0.707-2.505) *p* = 0.376	1.504 (0.535-4.225) *p* = 0.439	0.497 (0.174-1.421) *p* = 0.192
LDH per 100 U/L	1.179 (1.124-1.238) *p* <0.001	1.239 (1.183-1.297) *p* <0.001	1.183 (1.118-1.251) *p* <0.001	1.239 (1.134-1.353) *p* <0.001	1.258 (1.143-1.385) *p* <0.001
Ferritin per 200 ng/mL	1.014 (1.000-1.029) *p* = 0.051	1.005 (0.994-1.015) *p* = 0.378	1.014 (0.997-1.030) *p* = 0.099	0.997 (0.975-1.019) *p* = 0.773	1.037 (0.993-1.083) *p* = 0.099

Panel B (N = 2461)					
	Multivariate analysis				
**Variable**	**BMI < 25 N = 696 (28.28%) OR, 95% CI, *p*-value**	**25 ≤ BMI < 30 N = 785 (31.90%) OR, 95% CI, *p*-value**	**30 ≤ BMI < 35 N = 490 (19.91%) OR, 95% CI, *p*-value**	**35 ≤ BMI < 40 N = 261 (10.81%) OR, 95% CI, *p*-value**	**BMI ≥ 40 N = 224 (9.10%) OR, 95% CI, *p*-value**
Age < 65	0.271 (0.175-0.419) *p* <0.001	0.314 (0.217-0.456) *p* <0.001	0.353 (0.220-0.566) *p* <0.001	0.883 (0.476-1.638) *p* = 0.692	0.400 (0.194-0.824) *p* = 0.013
Age ≥ 65	3.696 (2.389-5.718) *p* <0.001	3.182 (2.193-4.616) *p* <0.001	2.836 (1.767-4.553) *p* <0.001	1.133 (0.611-2.102) *p* = 0.692	2.498 (1.213-5.142) *p* = 0.013
Male sex	0.978 (0.637-1.500) *p* = 0.919	1.107 (0.748-1.638) *p* = 0.612	1.322 (0.807-2.167) *p* = 0.268	1.033 (0.578-1.846) *p* = 0.914	0.747 (0.381-1.464) *p* = 0.395
WBC -10^3/μL	0.716 (0.418-1.227) *p* = 0.224	0.552 (1.308-1.992) *p* = 0.047	0.524 (0.149-1.841) *p* = 0.314	1.363 (0.373-4.988) *p* = 0.640	1.851 (0.716-4.782) *p* = 0.204
Neutrophils - 10^3/μL	1.445 (0.821-2.543) *p* = 0.203	1.940 (1.063-3.540) *p* = 0.031	2.021 (0.558-7.316) *p* = 0.284	0.745 (0.197-2.811) *p* = 0.664	0.550 (0.208-1.454) *p* = 0.228
Monocytes - 10^3/μL	1.405 (0.719-2.746) *p* = 0.320	0.957 (0.381-2.406) *p* = 0.925	1.686 (0.340-8.355) *p* = 0.522	0.466 (0.077-2.813) *p* = 0.405	0.141 (0.027-0.741) *p* = 0.021
Lymphocytes - 10^3/μL	1.410 (0.812-2.451) *p* = 0.223	1.864 (1.032-3.369) *p* = 0.039	1.997 (0.564-7.068) *p* = 0.284	0.342 (0.075-1.565) *p* = 0.167	0.452 (0.144-1.417) *p* = 0.173
LDH per 100 U/L	1.142 (1.068-1.220) *p* <0.001	1.256 (1.168-1.351) *p* <0.001	1.193 (1.100-1.294) *p* <0.001	1.097 (0.978-1.231) *p* = 0.115	1.251 (1.101-1.424) *p* <0.001
Ferritin per 200 ng/mL	1.015 (0.997-1.033) *p* = 0.105	0.997 (0.984-1.011) *p* = 0.680	1.046 (1.012-1.080) *p* = 0.007	1.007 (0.973-1.043) *p* = 0.681	1.049 (0.986-1.117) *p* = 0.130
Albumin - g/dL	0.388 (0.275-0.547) *p* <0.001	0.467 (0.324-0.672) *p* <0.001	0.642 (0.403-1.021) *p* = 0.061	0.687 (0.390-1.209) *p* = 0.193	0.521 (0.268-1.013) *p* = 0.055
D-dimer per 200 mg/mL	1.004 (1.000-1.008) *p* = 0.038	1.005 (1.000-1.010) *p* = 0.031	1.008 (1.002-1.014) *p* = 0.007	0.997 (0.989-1.006) *p* = 0.520	0.997 (0.989-1.004) *p* = 0.381
CRP per 20 mg/dL	1.079 (1.037-1.123) *p* <0.001	1.037 (0.999-1.076) *p* = 0.056	1.021 (0.975-1.068) *p* = 0.377	0.947 (0.881-1.018) *p* = 0.139	1.023 (0.951-1.100) *p* = 0.539
IL-6 per 20 pg/mL	*	*	*	*	*
Albumin - g/dL	0.571 (0.456-0.714) *p* <0.001	0.504 (0.400-0.634) *p* <0.001	0.617 (0.453-0.842) *p* = 0.002	0.619 (0.401-0.995) *p* = 0.030	0.523 (0.316-0.867) *p* = 0.012
D-dimer per 200 mg/mL	1.003 (1.001-1.006) *p* = 0.017	1.003 (1.000-1.005) *p* = 0.058	1.005 (1.002-1.009) *p* = 0.004	1.001 (0.997-1.005) *p* = 0.598	1.003 (0.998-1.009) *p* = 0.189
CRP per 20 mg/dL	*	*	*	*	*
IL-6 per 20 pg/mL	*	*	*	*	*

Panel C (N = 665)					
	Multivariate analysis				
**Variable**	**BMI < 25 N = 164 (24.66%) OR, 95% CI, *p*-value**	**25 ≤ BMI < 30 N = 204 (30.68%) OR, 95% CI, *p*-value**	**30 ≤ BMI < 35 N = 137 (20.60%) OR, 95% CI, *p*-value**	**35 ≤ BMI < 40 N = 78 (11.73%) OR, 95% CI, *p*-value**	**BMI ≥ 40 N = 82 (12.33%) OR, 95% CI, *p*-value**
Age < 65	0.565 (0.247-1.288) *p* = 0.174	0.271 (0.136-0.542) *p* <0.001	0.550 (0.225-1.343) *p* = 0.189	1.118 (0.365-3.422) *p* = 0.845	0.232 (0.066-0.807) *p* = 0.022
Age ≥ 65	1.771 (0.777-4.040) *p* = 0.174	3.684 (1.844-7.359) *p* <0.001	1.819 (0.745-4.445) *p* = 0.189	0.894 (0.292-2.737) *p* = 0.845	4.320 (1.240-15.052) *p* = 0.022
Male sex	1.120 (0.458-2.741) *p* = 0.804	0.948 (0.438-2.052) *p* = 0.892	0.779 (0.319-1.901) *p* = 0.583	1.151 (0.407-3.254) *p* = 0.791	0.331 (0.089-1.226) *p* = 0.098
WBC -10^3/μL	1.646 (0.293-9.234) *p* = 0.571	0.327 (0.053-2.030) *p* = 0.230	0.861 (0.097-7.632) *p* = 0.893	3.940 (0.154-100.864) *p* = 0.407	3.871 (0.599-25.011) *p* = 0.155
Neutrophils - 10^3/μL	0.607 (0.104-3.558) *p* = 0.580	3.276 (0.509-21.106) *p* = 0.212	1.203 (0.126-11.448) *p* = 0.872	0.239 (0.008-6.946) *p* = 0.405	0.242 (0.035-1.665) *p* = 0.149
Monocytes - 10^3/μL	0.817 (0.080-8.376) *p* = 0.865	0.925 (0.163-22.754) *p* = 0.603	3.080 (0.217-43.706) *p* = 0.406	0.288 (0.009-9.734) *p* = 0.488	0.117 (0.006-2.341) *p* = 0.160

Lymphocytes - 10^3/μL	0.574 (0.100-3.304) $p = 0.534$	1.795 (0.194-16.565) $p = 0.606$	1.238 (0.130-11.833) $p = 0.853$	0.201 (0.005-8.219) $p = 0.397$	0.076 (0.005-1.263) $p = 0.072$
LDH per 100 U/L	1.221 (1.021-1.460) $p = 0.028$	1.250 (1.087-1.437) $p = 0.002$	1.195 (0.994-1.436) $p = 0.058$	1.041 (0.868-1.248) $p = 0.667$	1.117 (0.898-1.388) $p = 0.320$
Ferritin per 200 ng/mL	1.065 (1.014-1.119) $p = 0.012$	1.001 (0.968-1.034) $p = 0.973$	1.088 (1.004-1.178) $p = 0.040$	1.174 (0.999-1.378) $p = 0.051$	1.351 (1.016-1.797) $p = 0.038$
Albumin - g/dL	0.313 (0.134-0.731) $p = 0.007$	0.938 (0.477-1.845) $p = 0.853$	0.611 (0.275-1.361) $p = 0.228$	0.747 (0.260-2.144) $p = 0.587$	0.606 (0.184-1.994) $p = 0.410$
D-dimer per 200 mg/mL	0.997 (0.989-1.006) $p = 0.535$	1.005 (0.998-1.013) $p = 0.152$	1.018 (1.000-1.036) $p = 0.048$	0.993 (0.977-1.009) $p = 0.364$	1.003 (0.994-1.013) $p = 0.499$
CRP per 20 mg/dL	0.990 (0.911-1.076) $p = 0.811$	1.022 (0.957-1.091) $p = 0.524$	1.046 (0.941-1.162) $p = 0.407$	0.956 (0.786-1.162) $p = 0.650$	1.022 (0.867-1.203) $p = 0.798$
IL-6 per 20 pg/mL	1.009 (0.996-1.022) $p = 0.179$	1.011 (0.998-1.024) $p = 0.088$	1.037 (0.992-1.085) $p = 0.109$	1.007 (0.974-1.040) $p = 0.693$	1.010 (0.987-1.034) $p = 0.401$

Notes: (1) BMI in kg/m2, (2) age in years, (3) Panel A refers to a cohort of 5068 patients that had available results for all these markers: WBC, neutrophils, monocytes, lymphocytes, LDH, ferritin, albumin, d-dimer, (4) Panel B refers to a cohort of 2461 patients that had available results for all the markers of Model A and CRP, (5) Panel C refers to a cohort of 665 patients that had available results for all the markers of Model B and IL-6.

Abbreviations: BMI = body mass index, g = gram, ng = nanogram, mg = milligram, L = liter, dL = deciliter, mL = milliliter, μL = microliter, U = unit, CRP = C-reactive protein, IL-6 = interleukin-6, LDH = lactic dehydrogenase.
